# The measurement of tissue interface pressures and changes in jugular venous parameters associated with cervical immobilisation devices: a systematic review

**DOI:** 10.1186/1757-7241-21-81

**Published:** 2013-12-03

**Authors:** Alison Sparke, Sarah Voss, Jonathan Benger

**Affiliations:** 1 Research Paramedic, Great Western Ambulance Service NHS Trust, Bristol, UK; 2 Research Fellow in Emergency Care, Faculty of Health and Life Sciences, University of the West of England, Bristol, UK; 3 Professor of Emergency Care, Faculty of Health and Life Sciences, University of the West of England, Bristol, UK

**Keywords:** Interface pressure, Cervical collar, Pressure ulcers, Jugular venous pressure, Intracranial pressure, Head injury

## Abstract

Cervical immobilisation is commonly applied following trauma, particularly blunt head injury, but current methods of immobilisation are associated with significant complications. Semi-rigid disposable cervical collars are known to cause pressure ulcers, and impede effective airway management. These collars may also exacerbate a head injury by increasing intracranial pressure as a result of external compression of the jugular veins. There is a clear imperative to find ways of effectively immobilising the cervical spine whilst minimising complications, and any assessment of existing or new devices should include a standardized approach to the measurement of tissue interface pressures and their effect on jugular venous drainage from the brain. This systematic review summarises the research methods and technologies that have been used to measure tissue interface pressure and assess the jugular vein in the context of cervical immobilisation devices. 27 papers were included and assessed for quality. Laboratory investigations and biomechanical studies have gradually given way to methods that more accurately reflect clinical care. There are numerous accounts of skin ulceration associated with cervical collars, but no standardised approach to measuring tissue interface pressure. It is therefore difficult to compare studies and devices, but a pressure of less than 30 mmHg appears desirable. Cervical collars have been shown to have a compressive effect on the jugular veins, but it is not yet certain that this is the cause of the increased intracranial pressure observed in association with cervical collar use. This is the first review of its type. It will help guide further research in this area of trauma care, and the development and testing of new cervical immobilisation devices.

## Introduction

Traumatic injuries as a result of road traffic collisions are becoming more prevalent worldwide, with over a million deaths annually, and this figure is predicted to rise [1]. In developed countries it is standard practice both in pre-hospital and in-hospital care to apply a semi-rigid cervical collar to the neck of patients presenting with blunt trauma, particularly head injury. These collars are applied with the aim of minimising neck movement prior to definitive clinical and/or radiological assessment, in order to reduce the risk of further damage that may result if unrestricted neck movement occurs in the presence of an unstable cervical spine injury. This practice is currently adopted by all ambulance trusts in the United Kingdom (UK), and promoted by the Advanced Trauma Life Support (ATLS) and similar courses [2].

However, the actual incidence of spinal cord injury is very low, at 10-15 per million population per year in the UK [3]. In the vast majority of patients semi-rigid cervical collars are fitted “as a precaution” in the absence of cervical spine injury; this means that most patients do not benefit from their application. Semi-rigid collars are often left in place for many hours, particularly in unconscious patients, with on-going use on intensive care and orthopaedic or neurosurgical wards. In a healthy and non-spinally injured patient continuous contact with the collar, and the resulting tissue interface pressures, can lead to early tissue breakdown and ulceration [4]. This is even more problematic in the presence of an actual spinal injury, due to lack of neural tone and decreased blood flow to the affected area [5]. As a result, the onset of ulceration and tissue breakdown is more rapid in patients where it is least desirable.

It seems likely that that the use of semi-rigid collars can exacerbate an existing traumatic brain injury [6-8]. This potential to exacerbate a head injury appears to occur as a result of increases in intracranial pressure (ICP) associated with the application of a semi-rigid collar, but is not well understood. Existing research suggests that increases in ICP are attributable to some degree of interaction with the jugular veins [9,10]. However, further work is required in this area.

Since most patients to whom a semi-rigid collar is applied do not need cervical immobilisation, and with such devices known to cause clinically significant complications, new procedures and devices are clearly required [11]. Research methods regarding cervical range of motion have recently been reviewed [12]; this should be taken into consideration when conducting a thorough evaluation of devices. In addition, any assessment of existing or new devices should include a standardized approach to the measurement of tissue interface pressure and the devices effect on jugular venous drainage from the brain. We therefore undertook a systematic review to summarise the evidence to date and inform further research in this area.

### Aim

This systematic review was conducted to identify and summarise research relevant to the measurement of tissue interface pressures and assessment of the jugular veins and intracranial pressure, with reference to cervical immobilisation devices. The review has two objectives:

a) To identify scientific methods used to measure tissue interface pressures occurring in association with cervical immobilisation devices and summarise the findings from the research;

b) To identify scientific methods used to research the jugular vein and intracranial pressure in association with cervical immobilisation devices and summarise findings from the research.

## Methods

### Data sources and search strategy

A systematic search of international literature was conducted in AMED (Allied and Complementary Medicine), BNI (British Nursing Index), EMBASE (biomedical and pharmacological), MEDLINE (biomedical and life sciences), and CINAHL (nursing, biomedicine and health sciences) databases. A qualified librarian assisted in planning and performing the searches. Boolean/phase mode was applied for two searches and the search strategy is shown in Appendix 1. It was necessary to conduct a key word search as there is no MeSH term for cervical collar or orthoses. The searches were restricted to papers published in the English language and were conducted during October 2012; the last access to the database was on 31^st^ October 2012.

### Inclusion and exclusion criteria

Scoping searches revealed that there were likely to be only a limited number of relevant randomised studies. Therefore, quasi-experimental and observational studies were eligible for inclusion. Due to the paucity of research in this area case reports and literature reviews were also included.

Articles were excluded if the primary focus of the paper was children, animals, chronic neck complaints or pressure ulcers caused by devices not designed to immobilise the cervical spine.

### Study selection

The study selection was a two stage process. Study titles and, where available, abstracts were initially screened by a single reviewer (AS) and first stage decision against the exclusion criteria was made. All relevant full-text articles were retrieved for detailed assessment by two reviewers (AS and SV) and those meeting the exclusion criteria were rejected. Studies relevant to the objectives were included in the final review.

### Quality assessment

Each paper was assessed for quality and the limitations of each study, including risk of bias, were considered. These are summarised in Tables 1 and 2. This area of research is currently in development and there is a lack of published research. None of the papers returned by the searches were large prospective randomised controlled trials assessing interface pressures or changes in jugular venous parameters associated with cervical immobilisation devices. Therefore, no formal risk of bias tools were used. However, it is important to fully consider the impact of any limitations to inform future investigations. For this reason all studies identified were included in the review and the various limitations of the studies are discussed in the results.

**Table 1:** Reviewed articles for tissue interface pressure

**Reference**	**Country**	**Article type & subjects**	**Observations**	**Key findings**	**Weaknesses**
			**Measurements**		
Jacobsen et al., 2008 [5]	USA	Literature review	Reduction in occipital pressure ulcers	Improved nursing education and a variety of collars caused an reduction in occipital ulcer incidence	Only looked at their own trauma centre
Liew et al., 1994 [13]	Australia	Case review: 2 patients	Pressure ulcer development	Ulceration is a complication of hard collar use	Only looked at 2 patients
Murphy et al., 1997 [14]	USA	Case review: 1 patient	Pressure ulcer development	Occipital pressure ulcers after collar use are common; improved wound care/more suitable collars advised	Only one patient reviewed
Walker, 2012 [4]	UK	Retrospective analysis:	Pressure ulcer development	Cervically immobilised patients have an increased risk of developing pressure ulcers	Looked at all types of immobilisation not just semi-rigid collars
		90 patients			
Powers, 1997 [20]	USA	Retrospective analysis	Pressure ulcer development	Improved education, more suitable collars plus early collar removal protocol resulted in a ulcer reduction	Only looked at their own trauma centre
Blaylock, 1996 [19]	USA	Retrospective analysis and study: 20 patients	Pressure ulcer development	Improved education on skin condition/wound care and collar fitting, plus a new collar design resulted in no ulcers in the subjects.	Possibility that the team were conscious regarding ulcer development. Small sample size and only one trauma centre included
Molano et al., 2004 [15]	Spain	Retrospective study:	Pressure ulcer development	23.9% had ulcers; with occipital ulcers being more problematic to treat	Only looked at their own unit
		92 patients			
Chendrasekhar et al., 1998 [21]	USA	Retrospective study:	Pressure ulcer development	Ulceration is related to duration of collar wear; early collar removal advocated	Only 34 patients actually included due to mortality and only 8 had their collar removed earlier than normal
		52 patients			
Beavis, 1989 [17]	UK	Study: 10 volunteers,	Tissue Interface Pressure at chin and occiput	Passive and active results showed wide variance, but Beavis felt that the pressure was a positive feature, being an incentive not to move	No consideration for the impact of high interface pressure
		4 collars			
			(25-172 mmHg)		
Black et al., 1998 [22]	USA	Study: 20 volunteers,	Tissue Interface Pressure at occiput (39-83 mmHg) plus skin temperature and humidity	No difference between the interface pressures of the collars was found; felt skin humidity was an important factor in ulcer development	Collars were worn for up to 30 minutes possibly impacting on pressure readings due to foam compression within the collar
		2 collars			
Ferguson et al., 1993 [10]	UK	Study: 5 patients,	Tissue Interface Pressure around neck area (1.2-11.8 mmHg)	Pressures recorded depended on the tightness of the collar	Sensor positions were possibly not indicative of true pressure points; tensions applied were subjective
		6 collars			
Fisher, 1978 [16]	USA	Study: 8 patients,	Tissue Interface Pressure at chin an d occiput	Passive interface pressures varied with collar tightness:	Used the same person to apply the collar; ‘tightness’ was subjective
		1 collar			
			(25-105 mmHg)		
Plaisier et al., 1994 [18]	USA	Study: 20 volunteers,	Tissue Interface Pressure at chin, occiput and mandible (27-57 mmHg); plus comfort	Philadelphia/Stifneck collars exceed capillary closing pressure in some positions, yet Newport/Miami J did not; comfort ratings tallied with this	Skin humidity temperature data would have confirmed their ‘skin friendly’ endorsement of the Newport and Miami J collar
		4 collars			
Tescher et al., 2007 [23]	USA	Study: 48 patients,	Tissue Interface Pressure at occiput and mandible	Miami J/Miami J with Occian back had lower pressures recorded both seated and supine; however all maximal pressures recorded exceeded capillary closing pressure	Admit that interface pressure is an important consideration in ulcer development but admit other factors may play an important part
		4 collars			

**Table 2:** Reviewed articles for jugular vein dimension and intracranial pressure (ICP)

**Reference**	**Country**	**Article type & subjects**	**Observations**	**Key findings**	**Weaknesses**
			**Measurements**		
Craig et al., 1991 [25]	UK	Case review:	Intracranial pressure and collar use	Collar use was associated with impaired venous drainage and as such relates to an increase in ICP	Only two patients
		2 patients			
Lemyze et al., 2011 [28]	France	Case review:	Level of consciousness	Collar use post hanging may exacerbate cerebral oedema due to compression of neck veins	Only one patient
		1 patient			
Dunham et al., 2008 [32]	USA	Literature Review	Risks with MRI and collar use	Collar use is associated with a rise in ICP, and secondary brain injury; early collar removal advocated	Evidence supports the theory but no substantial proof
Ho et al., 2002 [7]	China	Literature Review	Intracranial pressure and collar use	Collars appear to act like a tourniquet around the neck, potentially exacerbating a head injury and increasing ICP	Evidence supports the theory but no substantial proof
Dunham et al., 2011 [8]	USA	Simulation study	Collar use and outcome	Early collar removal is advocated for unstable/high risk and stable patients with spinal injuries	Simulation study only; no real proof for claims
Davies et al., 1996 [6]	UK	Study:	ICP before and after collar application	Stifneck collar may cause a rise in intracranial pressure.	Supports hypothesis for collars affecting ICP but exact mechanism for rise in ICP was not determined
		19 patients			
Hunt et al., 2001 [29]	UK	Study:	ICP before and after collar application	Collars are associated with a rise in intracranial pressure, potentially worse if the ICP is higher to start with	Supports hypothesis for collars affecting ICP but exact mechanism for rise in ICP was not determined
		30 patients			
Kolb et al., 1999 [24]	USA	Study:	Cerebrospinal fluid pressure before and after collar application	Cerebrospinal fluid pressure increased after collar application, but no clear associated with BMI	Collars were placed when the patient was inclined to the side rather than supine; exact mechanism for rise in ICP was not determined
		20 patients			
Kuhnigk et al., 1993 [30]	Germany	Study:	Intracranial pressure and collar use	Found no correlation between collar use and increased intracranial pressure	Baseline ICP readings were higher than in other reports and as such may have had an impact on the results
		18 patients			
Mobbs et al., 2002 [9]	Australia	Study:	ICP before and after collar application	Intracranial pressure was higher following the application of a collar	Supports hypothesis for collars affecting ICP but exact mechanism for rise in ICP was not determined
		10 patients			
Stone et al., 2010 [31]	USA	Study:	Jugular vein dimensions before and after collar application	Collar application associated with alteration to jugular vein diameter indicative of venous obstruction	Supports hypothesis for collars affecting ICP but no mechanism was determined as actual ICP was not measured
		42 volunteers			
Porter et al., 1999 [27]	UK	Study:	ICP before and after collar application	All patients showed a rise in ICP following collar application	Supports hypothesis for collars affecting ICP but no mechanism was determined
		9 patients			
Ferguson et al., 1993 [10]	UK	Study:	Tissue Interface Pressure around neck area	Normal jugular venous pressure when supine was 2-7 mmHg, concluded pressures exerted on the neck over this will cause a ‘back pressure’	Supposition rather than fact with the relationship of collar use to increased ICP
		5 patients			
Raphael et al., 1994 [26]	UK	Study:	Cerebrospinal fluid pressure before and after collar application	7/9 experienced raised cerebrospinal fluid pressure following collar application	Supports hypothesis for collars affecting ICP but no mechanism was determined
		9 patients			

## Results

116 articles were returned from the search. 81 were excluded in the first stage of assessment and a further 7 during the second stage. 27 papers were included in the review.

**Figure 1:**
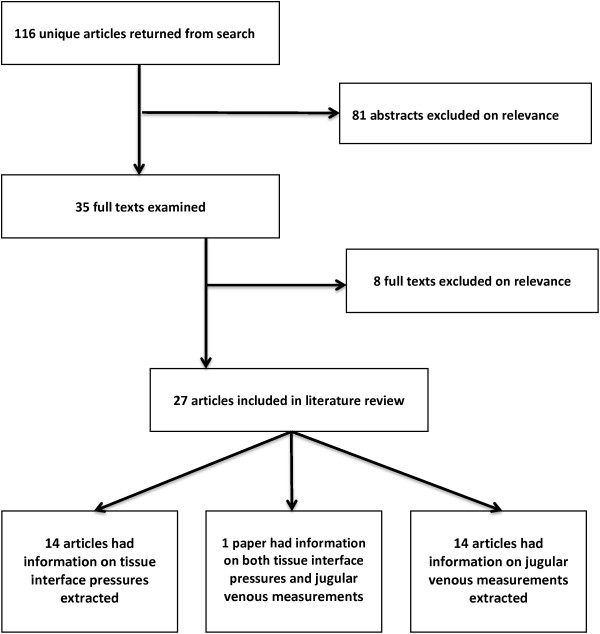
Flow of studies included in the review.

Information was extracted from each study on tissue interface pressures (14 papers included [4,5,10,13-23]) and jugular venous measurements (14 papers included [6-10,24-32]) in association with cervical immobilisation devices (Figure 1). One paper [10] was included in both searches.

### Research and scientific methods to measure tissue interface pressures

There have been a number of observational, retrospective studies to document cases of skin breakdown and ulceration resulting from semi-rigid cervical collars. The 14 papers included in this section of the review are summarised in Table 1.

In 1994 Liew et al carried out a case review of two multi-trauma patients [13], noting that they had developed significant occipital pressure ulceration as a result of cervical collar use, whilst Murphy, in 1997 [14], was the first to note that *‘development of occipital pressure ulcers under cervical collars is a common problem’* (Pg. 60)*.* Molano et al also identified pressure sores as a complication of collar use following a review of patients admitted with an acute cervical spine injury [15]. Pressure sores developed most frequently at occipital, chin and supra-scapular zones; with occipital injuries the most severe.

Jacobsen et al determined that whilst only 1% of all occipital pressure ulcers are attributable to the use of a rigid cervical collar [5], the incidence of hospital acquired pressure ulcer ranges from 23.9% to 44%. They also noted that collar-related pressure ulcers may develop at various locations on the body; but occipital pressure ulcers are the most serious because there is very little subcutaneous tissue over the bone, confirming the work of Molano et al [15]. Jacobsen et al also drew attention to cases where the application of cervical collars exerted a pressure greater than the capillary closing pressure, thus restricting blood flow to contact areas, exacerbating the problem. A retrospective case review by Walker identified immobilised patients as being at greatest risk of pressure ulceration exacerbated by immobilisation devices [4]. This resulted in prolonged recovery times and additional healthcare dependence. He recommended that practitioners be aware of the high risk of pressure ulcer development in patients with a cervical orthosis, especially if they are not able to relieve the pressure or verbalise discomfort. Walker also considered the factors that affect development of pressure ulcers in the spinally injured immobilised patient, such as reduced mobility, use of immobilisation devices, malnutrition and hypoperfusion.

Following the observation that skin breakdown and ulceration are common after the application of a cervical collar, the next logical step became measurement of the tissue interface pressures that occur.

Fisher observed that the resting tissue interface pressure (TIP) exerted by a sternal occipital mandibular immobilisation (SOMI) orthosis depended on how tight the orthosis was fitted (pressures of between 25 and 105 mmHg were recorded) [16]. However, when Beavis [17] and Ferguson et al [10] investigated TIP using pneumatic pressure sensors placed on the necks of volunteers, they obtained varied results. Ferguson et al recorded mean TIPs of between 0.3 and 11.8 mmHg from passive supine participants, whilst Beavis recorded TIPs of between 55 and 150 mmHg for active movements of flexion and extension with seated participants. The findings from these studies indicate that there is considerable variation in TIP measurements depending on the testing conditions and subject movement, with a lack of standardization in the testing protocols used.

Plaisier et al carried out a study comparing four cervical collars (Stifneck®, Miami J, Philadelphia and Newport) [18]. Tissue interface pressures were measured using electro-pneumatic sensors at the chin, occiput and mandible, and opinions on comfort noted. All collars were tested on healthy volunteers in both a seated and supine position (adopting features from the work of Beavis and Ferguson et al). TIP exerted by the Stifneck® collar was noted to exceed capillary closing pressure (CCP); this also applied to the Philadelphia collar, particularly when wearers were supine. The Miami J and Newport exerted a TIP well below CCP. Newport and Miami J devices were therefore perceived as ‘skin friendly’, due to comfort and low TIP readings, with the conclusion that they may reduce the incidence of soft tissue complications and improve patient compliance.

Blaylock conducted a retrospective analysis of pressure ulcer development as well as a clinical trial of a new collar in 20 patients [19]. The recommended outcomes were for improved skin care, education on proper collar fitting and choice of collar, as well as implementing the new collar for trauma patients. A multidisciplinary approach to reducing complications associated with collar use was also adopted by Powers [20]. By improving nursing care, using a different type of collar and developing a new protocol for cervical spine clearance, Powers was able to eliminate the incidence of pressure ulcers related to collar use from his department. A key outcome from this work was that patients were wearing collars for a much shorter period of time. A recommendation for early collar removal was also the conclusion of a study by Chendrasekhar et al [21]. They examined patients with severe closed head injury; 13 (34%) developed occipital pressure sores related to cervical collar use, whilst those who underwent early collar removal did not.

Black et al evaluated skin breakdown in healthy volunteers with two collars (Aspen and Philadelphia). They measured occipital pressure using the Talley digital skin pressure evaluator, Model SD500 (Talley Group Ltd, England); as well as skin temperature and humidity [22]. Pressure and skin temperature were comparable between the collars, but skin humidity increased with the Philadelphia collar, increasing the risk of pressure ulcer development. Tescher et al carried out a similar study looking at the TIP exerted by four cervical collars (Miami J, Aspen, Philadelphia and Miami J) [23]. Interface pressures were measured at the occiput and the anterior mandible using the XSensor X2 (XSENSOR Technology Corporation, Canada) system of capacitance pressure-sensing transducers, in both a seated and supine position, similar to Plaisier. They showed that the TIP exerted by the collar at the mandible varied greatly (between 22.08 and 70.89 mmHg); but more significantly they noted capillary closing pressure was only 32 mmHg. As the TIP sites were different it is difficult to compare these results to other studies, however Tescher does confirm Black’s theory that moisture may impact on pressure sore development. Tescher also identified a number of other important factors that influence TIP. These include BMI, fluid retention due to the traumatic inflammatory response, poorly fitting collars and shear forces exerted on the skin.

### Research and scientific methods to assess the jugular vein and intracranial pressure

There has been a limited amount of work to assess the jugular vein and its relationship to intracranial pressure (ICP) when a cervical collar is applied. The link between the use of rigid cervical collars and an increase in intracranial pressure (especially in head injured patients) is widely considered as fact, yet the exact aetiology is unknown. The 14 papers included in this section of the review are summarised in Table 2.

A number of studies have postulated a venous tourniquet effect from a rigid cervical collar, with an impact on intracranial pressure [10,24]. This has been supported by research demonstrating that ICP increases when a cervical collar is applied, and then gradually declines on removal. The most obvious explanation is an increase in intracranial blood volume due to pressure on the neck impairing venous drainage [6,25-28].

Hunt et al [29] examined the effect of cervical collars on patients with a traumatic brain injury. Results showed a rise in baseline intracranial pressure when collars were applied. There were no other changes to cardio-respiratory parameters; therefore venous compression in the neck was considered to be the likely cause of the observed effect. Moreover, they showed that the most significant increases in intracranial pressure were in patients with a higher baseline to start with, suggesting that this effect of cervical collars is more pronounced in patients with a greater risk of neurological deterioration. These findings were confirmed by Mobbs et al who concluded that early assessment of the cervical spine in a head injured patient is essential [9]. They also postulated that obstruction of venous drainage and the potential for the collar to cause a painful stimulus may both impact on ICP. However, they were unable to determine the exact reason for the observed increase in ICP.

Kuhnigk et al measured ICP in 18 severely head injured patients before and after placement of a collar [30], but did not find a significant change in ICP following collar application. They therefore concluded that the risk of increasing ICP in a head injured patient was low. These findings contradict the other papers reviewed, and are unexplained. However, the work was carried out in patients with a higher baseline ICP than the other studies, and this may have been a factor.

Stone et al were the first to actually assess the effect of a cervical collar on the dimensions of the internal jugular veins, using ultrasound examination [31]. In healthy volunteers, they were able to show an increase in cross-sectional area following the application of a cervical collar, supporting the hypothesis that collars create venous obstruction in the neck. It seems likely that this obstruction contributes to an increase in intracranial pressure, but this could not be confirmed because the measurement of ICP is invasive, and therefore not suitable for volunteer studies.

A literature review by Ho et al noted that between 25% and 75% of all traumatic cervical spine injuries are unstable, and therefore recommended cervical stabilisation [7]. However, they hypothesised that a cervical collar may act as a tourniquet around a patient’s neck, mirroring Ferguson’s concerns. They therefore recommended rapid clearance of the cervical spine in trauma patients to reduce the risk of raised intracranial pressure. Prolonged collar use was also the focus of a literature review by Dunham et al [32]. The risk of raised ICP due to collar use was found to be 35.8% in unstable patients, and they also noted that a cervical collar was associated with increased intracranial pressure in patients without head injury. In a later simulation study, Dunham et al showed that early collar removal was associated with more favourable outcomes in both unstable and stable cervical spine injured patients [8].

## Discussion

This systematic review has identified relatively few studies that assess the impact of a semi-rigid cervical collar on tissue interface pressures or jugular venous drainage from the brain. Those that have been done tend to use small samples of volunteers with variable methodologies and testing protocols, which makes comparison between papers difficult.

The evidence that is available demonstrates an important causal relationship between the use of semi-rigid cervical collars and the development of pressure ulcers, as a result of the skin pressure exerted at key tissue interfaces. A number of retrospective studies have linked cervical collars to the development of pressure ulcers, but few have actually determined the TIP exerted by various immobilisation devices. For those that do record TIP the results vary widely, ranging from 0.3 mmHg to 150 mmHg, and this makes it difficult to draw any clear conclusions or compare different devices. Much of the variation is likely to be due to differences in test conditions (e.g. seated versus supine) and the technical characteristics of the devices used to measure TIP, as well as actual differences between the immobilisation devices under test. Future research in this area would benefit from the development and implementation of standardised TIP measurement techniques and protocols.

With respect to jugular vein assessment, we found a number of studies demonstrating an association between the use of semi-rigid cervical collars and increased intracranial pressure (ICP), though this was not universal. There is, however, very little research to determine the underlying cause. It seems likely that existing cervical collars have a tendency to obstruct jugular venous drainage, by acting as a tourniquet around the neck, but although it has been shown that collars have an effect on the dimensions of the internal jugular vein [30], a causal link to increased ICP has yet to be demonstrated. Other factors, such as discomfort or pain associated with collar use, may also be important, and further work is required in this area.

## Conclusions

There were some limitations to this review. The first stage of the selection process was carried out by a single reviewer; however the exclusion criteria were clearly defined and this is unlikely to have influenced the end result. In addition, it was not possible to use a formal tool to assess risk of bias; thus the papers discussed in the results are of varying validity. Nonetheless, in light of the global use of cervical immobilization devices in daily practice, these findings indicate that further scientific scrutiny of the complications described is warranted.

Tissue interface pressure (TIP) is an important factor in the use of cervical immobilisation devices, and to prevent the development of skin ulcers the early removal of a collar is recommended. However, this is not always possible, and where cervical immobilisation is required for a prolonged period of time measures should be taken to reduce the pressure exerted by a collar or similar device. There is no standardised approach to measuring TIP, making it difficult to compare studies and cervical immobilisation devices; however it is reasonable to aim for TIPs of less than 30 mmHg, since this approximates to the capillary closing pressure.

The relationship between cervical collars, the jugular vein and intracranial pressure (ICP) requires further research. Evidence to date demonstrates a strong relationship between cervical collars and altered jugular vein dimensions. This is likely to be the cause of the raised ICP associated with collar application, but has yet to be confirmed experimentally. Research that simultaneously assesses the jugular vein and ICP is therefore required.

New approaches to cervical immobilisation, which avoid these common complications of the collars currently in use, would be welcome.

## Appendix 1

Search One (tissue interface pressures):

1. cervical collar

2. cervical orthos*

3. hard cervical collar

4. collar

5. #1 OR #2 OR #3 OR #4

6. craniofacial

7. occip*

8. scalp

9. chin

10. mand*

11. #6 OR #7 OR #8 OR #9 0R #10

12. contact

13. interface

14. tissue interface pressure

15. pressure

16. ulcer

17. pressure ulcer

18. #12 OR #13 OR #14 OR #15 OR #16 OR #17

19. 5 AND 11 AND 19

Search Two (jugular veins and intracranial pressure):

1. cervical collar

2. cervical orthos*

3. hard cervical collar

4. collar

5. #1 OR #2 OR #3 OR #4

6. jugular vein

7. jugular pressure

8. jugular vein diameter

9. jugular vein measurements

10. head injury

11. neck veins

12. intracranial pressure

13. #6 OR #7 OR #8 OR #9 0R #10 OR #11 or #12

14. 5 AND 13

## Competing interests

The authors declare that they have no competing interests.

## Authors’ contributions

AS and SV conducted the review and drafted the manuscript. JB supervised the work, editing the manuscript and providing the clinical context. All authors edited and approved the final manuscript.
